# Design of a randomized controlled study of a multi-professional and multidimensional intervention targeting frail elderly people

**DOI:** 10.1186/1471-2318-11-24

**Published:** 2011-05-14

**Authors:** Katarina Wilhelmson, Anna Duner, Kajsa Eklund, Gunilla Gosman-Hedström, Staffan Blomberg, Henna Hasson, Helena Gustafsson, Sten Landahl, Synneve Dahlin-Ivanoff

**Affiliations:** 1 Vårdalinstitutet, The Swedish Institute for Health Sciences, University of Gothenburg and Lund, Sweden; 2 Department of Public Health and Community Medicine/Social Medicine, Institute of Medicine, The Sahlgrenska Academy at University of Gothenburg, Gothenburg, Sweden; 3 Department of Social Work, University of Gothenburg, Gothenburg, Sweden; 4 Department of Clinical Neuroscience and Rehabilitation, The Sahlgrenska Academy at University of Gothenburg, Gothenburg, Sweden; 5 School of Social Work and Welfare Studies, Lund University, Lund, Sweden; 6 Department of Business Administration, Lund University School of Economics and Management, Lund, Sweden; 7 Department of Physiology, Institute of Neuroscience and Physiology, The Sahlgrenska Academy at University of Gothenburg, Gothenburg, Sweden

## Abstract

**Background:**

Frail elderly people need an integrated and coordinated care. The two-armed study "Continuum of care for frail elderly people" is a multi-professional and multidimensional intervention for frail community-dwelling elderly people. It was designed to evaluate whether the intervention programme for frail elderly people can reduce the number of visits to hospital, increase satisfaction with health and social care and maintain functional abilities. The implementation process is explored and analysed along with the intervention. In this paper we present the study design, the intervention and the outcome measures as well as the baseline characteristics of the study participants.

**Methods/design:**

The study is a randomised two-armed controlled trial with follow ups at 3, 6 and 12 months. The study group includes elderly people who sought care at the emergency ward and discharged to their own homes in the community. Inclusion criteria were 80 years and older *or *65 to 79 years with at least one chronic disease and dependent in at least one activity of daily living. Exclusion criteria were acute severely illness with an immediate need of the assessment and treatment by a physician, severe cognitive impairment and palliative care. The intention was that the study group should comprise a representative sample of frail elderly people at a high risk of future health care consumption. The intervention includes an early geriatric assessment, early family support, a case manager in the community with a multi-professional team and the involvement of the elderly people and their relatives in the planning process.

**Discussion:**

The design of the study, the randomisation procedure and the protocol meetings were intended to ensure the quality of the study. The implementation of the intervention programme is followed and analysed throughout the whole study, which enables us to generate knowledge on the process of implementing complex interventions. The intervention contributes to early recognition of both the elderly peoples' needs of information, care and rehabilitation and of informal caregivers' need of support and information. This study is expected to show positive effects on frail elderly peoples' health care consumption, functional abilities and satisfaction with health and social care.

**Trial registration:**

ClinicalTrials.gov: NCT01260493

## Background

The elderly population is increasing, in Sweden as well as in many other countries [1], a trend which is expected to continue [2]. Increasing age often implies increasing frailty, and the oldest old are often described as a frail group. Frail elderly people are at high risk of developing chronic disease, multi-morbidity and functional impairments, which often result in dependence in daily activities [3-7].

Frailty has been recognized as a concept to describe a geriatric syndrome attributable to the multi-system deterioration of the reserve capacity at older ages [8]. The most frequently included characteristics are: mobility, balance, muscle strength, motor processing, cognition, nutrition, endurance and physical activity [3]. Frailty implies a risk of multi-morbidity and thereby a need of care from many care levels and from caregivers with different competences, such as gerontology, geriatrics, internal medicine, rehabilitation, nursing and social work. This makes it clear that frail elderly people need integrated, coordinated care [9].

Integrated care programmes have been used internationally to reduce fragmentation and to improve the continuity and coordination of care. Several programmes have shown positive effects, but it is not clear which components or interventions are essential to these programmes [10]. A review of randomised controlled studies of integrated care programmes for the frail elderly showed that five out of eight studies had positive effects on the elderly person and none had negative effects. Positive effects were reported on medication, client satisfaction, activity of daily living, quality of life and depression [11].

One important component in many of the integrated care programmes is case management. Case management was first implemented in psychiatric care [12,13]. It has also been used to coordinate the care of the elderly [12,14]. Case management has mostly been used in the US, UK, Australia and Italy. Owing to heterogeneity in study design, intervention content, outcome variables and population, the studies are difficult to compare. However, the effects seem to be mainly positive. Some studies have shown positive effects on both the individual and health care consumption, while others have failed to detect any effects [12,14].

Another way to enhance the continuity and integration of the health care of the elderly is geriatric screening and multidimensional assessment at the emergency ward, which has been introduced in many countries. It can be incorporated into practice without too much difficulty. It meets with a high level of acceptance, involves different categories of caregivers and improves the communication between them [15]. Previous research has found that interventions including geriatric nursing assessment and home based services results in functional benefits for elderly high-risk patients. Other key features of trials showing improvement is the selection of patients at high risk of adverse outcomes. One effective strategy is to keep the screening at the emergency ward brief and moving more of the intervention to the patient's home [16]. A systematic review identified that when a multidisciplinary comprehensive assessment was combined with an individually tailored intervention, this promoted functional activity, well-being and life satisfaction [17]. According to a meta-analysis, such interventions are able to decrease readmissions to hospital [18].

In Sweden, "health care chains" have become an important part of integrated health care [19]. A health care chain can be defined as coordinated activities in the health care system, linked together to achieve a final result of good quality for the patient [20]. A well-functioning care chain implies that the care is seen as a continuum running between different caregivers and care levels, and that one caregiver of high quality is not enough to create good care. Elderly frail people meet many different caregivers [21]. Often, the elderly person and their relatives are the only common link between the different care levels [22]. Previous studies have shown that elderly people and their relatives are seldom aware of the different caregivers' responsibilities, and that they have difficulties in knowing to whom turn to concerning different needs [23]. Many relatives experience that they have no influence on the care [24]. In order to achieve a continuum of care, the elderly people and their relatives must be involved in the planning, decision-making and performance of the care [15,25]. A review of randomised controlled studies of integrated care programmes for the frail elderly showed that the two studies that found the most client benefit were ones in which the elderly person was involved [11]. A review of interventions to prevent disability in frail community-dwelling older people points out promising features of interventions to be, for example, multidisciplinary and multi-factorial, individualized assessment and intervention, case management and long-term follow up [26].

The findings from the above-mentioned studies guided us in the design of a multi-professional and multidimensional intervention for frail community-dwelling elderly people, "Continuum of Care for Frail Elderly People". The content of the intervention has been elaborated in collaboration with representatives of the different care levels, i.e. emergency ward, department of geriatrics, department of internal medicine, municipal health and social care, and primary care. The design of the study includes quantitative and qualitative analyses of the effects of the intervention programme as well as of the implementation process, which is followed throughout the whole intervention period.

### Aims and hypothesis

The hypothesis is that this intervention programme for frail elderly people can reduce the number of visits to the emergency ward, increase the satisfaction with health and social care and maintain functional abilities. The overall aim of the study was to implement the intervention and thereby create a continuum of care for frail elderly people, from the emergency ward to their own homes, resulting in a better quality of care and higher cost-effectiveness. Another aim was to study the implementation process of the intervention programme.

This paper presents the study design, the intervention, the outcome measurements and the baseline characteristics of the study participants in accordance with the CONSORT recommendations for reporting pragmatic randomised controlled trials [27].

## Methods/Design

### Project context

The study is part of the research programme "Support for frail elderly persons - from prevention to palliation" (http://www.vardalinstitutet.net) which consists of three different interventions addressing frail elderly people in different phases of the disablement process, from pre-frail to very frail. These interventions address different requirements that arise during the aging process, ranging from health promotion to increasing needs of medical care, nursing, rehabilitation, social care and services and eventually the need of palliative care to promote symptom relief, quality of life, security and satisfaction with care during the final period of life.

The intervention "A continuum of care for frail elderly people" takes place in the municipality of Mölndal, Sweden, including municipal health and social care, the hospital of Mölndal, and primary care. Mölndal is a city situated on the west coast of Sweden, close to the city of Gothenburg. It had nearly 60,000 inhabitants at the beginning of 2009. The population of people aged 65-79 years was 6,289 persons at the beginning of 2009, and the population aged 80 and over was 2,592. In June 2008, 11.6% of the those aged 65 or older received some kind of help or care from the municipality. Mölndal Hospital is part of Sahlgrenska University Hospital, and includes, among others, an emergency ward and departments for internal medicine, geriatrics and orthopaedic care. Sahlgrenska University Hospital has 2300 beds in 165 wards. Twenty-six of these wards are located at Mölndal Hospital. This study includes patients discharged from the emergency ward, internal medicine and geriatrics.

### Study design

The study has a descriptive analytical and experimental design. The intervention is performed as a randomised controlled trial. The participants were randomised to two study arms, one intervention group and one control group. The implementation process is studied and analysed along with the intervention. Explorative interviews are performed with staff and study participants in order to gain an understanding of the intervention and its significance as well as of the implementation. Ethical approval was obtained for the study, ref. no: 413-08, Regional Ethical Review Board in Gothenburg.

### Study population

The study group includes 161 elderly people who sought care at the emergency department at Mölndal Hospital during the period October 2008 to June 2010 and who were discharged to their own homes in the municipality of Mölndal. Inclusion criteria were age 80 and older *or *65 to 79 with at least one chronic disease and dependent in at least one activity of daily living. Exclusion criteria were acute severe illness with immediate need of assessment and treatment by a physician (within ten minutes), dementia (or severe cognitive impairment), and palliative care. The intention was that the study group should comprise a representative sample of frail elderly people at a high risk of future health care consumption.

### Intervention group

The intervention involve collaboration between a nurse with geriatric competence at the emergency ward, the hospital wards and a multi-professional team for care of the elderly with a case manager in the municipality. The multi-professional team includes professionals with university degrees in nursing (the case manager), social work, occupational therapy and physiotherapy. The aim is to create a continuum of care from the emergency department, through the hospital ward to the elderly person's own home. In addition, there is support for relatives, initiated as early as at the hospital.

At the emergency ward, the nurse with geriatric competence made an assessment of the elderly patient's needs of rehabilitation, nursing, geriatric and social care. This assessment was transferred to the ward and to the case manager in the municipality. The case manager is responsible for contacting the ward and the patient in order to initiate discharge planning. Discharge planning is done in collaboration between the case manager, a social worker, the patient, and the nurse and physician in charge at the ward. Patient care planning is done in the elderly person's home within a couple of days after discharge. Patients discharged directly from the emergency ward were offered patient care planning by the case manager and the team. The multi-professional team is responsible for the patient care planning, which is done by involving the patient throughout the intervention. The care planning is based on a comprehensive geriatric assessment done by the team, followed up after one week by the case manager, and then at least every month. The elderly person is included in the intervention for at least one year.

The case manager contacts the relatives/informal caregivers, if approved by the elderly person, to give information/involve them in the planning and to offer them support and advice. This is initiated as soon as possible, often as early as when the elderly person is in the hospital.

### Control group

The control group receives conventional care and follow up. Access to a case manager or multi-professional team is not part of the present organization of municipal care for elderly persons living in Mölndal. When needed, the patient care planning is done at the hospital by a team from the community consisting of different professional groups (social worker, nurse and occupational therapist or physiotherapist) responsible for all care planning at the hospital. After discharge, another team from municipality elderly care - known as the district team - is responsible for the follow-up of the care planning. If the patient is discharged from the emergency department directly to their home, there is no routine for information transfer from the hospital to the municipality. In addition to conventional care, there are also assessments at the research follow ups for the control group - the same as for the intervention group, see under procedures below. If unmet needs are revealed at these research follow-ups, the elderly person will get advice on where and how to seek help.

### Procedure of the intervention study

The participants were recruited at the emergency wards. The nurse with geriatric competence screened most of the patients during her work shift (daytime, weekdays, approximately 3-4 days per week) to see if they fulfilled the inclusion criteria. If so, the nurse informed them about the study both verbally and in writing. The information included a description of the study, how it would be conducted and what was expected of people who agreed to participate. There were opportunities to ask questions if anything was unclear. It was stressed, both in the verbal and the written information that participation was voluntarily. Of all those invited to participate, 17 were invited by letter, as they had been discharged before the nurse was able to ask them. People who accepted to participate in the study were randomised to intervention or control by using a system of sealed opaque envelopes. All participants signed a written consent form. The study started with a pilot study to test intervention, inclusion/exclusion criteria and logistics. The pilot study comprised the first ten included participants.

A baseline interview and assessment were done within a week of discharge. In some cases it was not possible to do the baseline interview so soon, mostly because the frail elderly person not having enough strength. Follow-up data are collected at 3, 6 and 12 months, see table 1 for description of the objectives, outcome measures and follow ups of the study. On the follow ups, there was also sometimes a delay, owing to the frail elderly person's lack of strength or readmission to hospital. The baseline interviews for the intervention group were done by the multi-professional team as part of their comprehensive geriatric assessment. The baseline interview for the control group and all follow ups for both groups were done by research assistants, who were occupational therapists, nurses or social scientists. The interviews were performed in the participants' home. All interviewers were well trained in interviewing, assessing and observing, according to the guidelines for the different outcome measurements. It was not possible to keep the interviewer blinded to group assignment when doing the follow ups. The reasons for this are threefold: 1) in most cases the participant revealed the assignment unintentionally; 2) some elderly people were not aware that the case manager was part of the intervention and thus did not answer the questions about their experience of receiving the intervention unless the research assistant knew that they were assigned to the intervention; and 3) we assumed there would be less attrition if the elderly person could meet the same research assistant for most of the follow ups.

**Table 1:** Outcome measures and follow ups

Primary Outcome	Measurement	T0 baseline	T1 3 months	T2 6 months	T3 1 year
Health care consumption	Register data				
Satisfaction with health and social care	Questionnaire	X	X	X	X
Functional ability	The Berg Balance Scale	X	X	X	X
	Gait speed four-meter walking test	X	X	X	X
	Grip strength: North Coast dynamometer	X	X	X	X

**Secondary Outcome**	**Measurement**				

Fatigue	Tiredness scale	X	X	X	X
Physical activity	Questionnaire	X	X	X	X
	Physical and domestic activity scale	X	X	X	X
Activities of daily living	The ADL staircase	X	X	X	X
Weight loss	The Göteborg Quality of Life Instrument	X	X	X	X
Cognition	Mini mental State Exam (MMSE)	X		X	X
Visual impairment	KM visual acuity chart	X		X	X
Depression	GDS 20	X	X	X	X
Health-related quality of life	EQ5D	X	X	X	X
Life satisfaction	Fugl-Meyer - LiSat	X	X	X	X
Participation/leisure activities	Questionnaire	X	X	X	X
Social support	Questionnaire	X	X	X	X
Falls	Questionnaire	X	X	X	X
Fear of falls	FES-I	X	X	X	X
Decisional autonomy	Impact on Participation and Autonomy Questionnaire	X	X	X	X
Self-rated health	SF 36 (one question)	X	X	X	X
Illness	CIRS-G	X		X	X
Symptoms	The Göteborg Quality of Life Instrument	X	X	X	X
Mortality	Register data				

Meetings are held regularly with all personal in the intervention from one month before starting the inclusion process and throughout the entire intervention period (including the pilot study). In addition, the project leaders for the research and the different care levels, i.e. emergency care, municipal care and primary care, meet regularly during the intervention period.

## Research questions and outcome measures

There are two overarching research questions:

1) Can an intervention for frail elderly people at risk of high care consumption

• decrease health care utilization?

• maintain/increase functional abilities, activities of daily living, health related quality of life and life satisfaction?

• increase satisfaction with rehabilitation, health and social care for the frail elderly and their relatives?

• be cost-effective?

2) Which obstructing and facilitating components can be identified when implementing the intervention

• on the operative level, concerning the actions of the professionals involved?

• on the operative and management level regarding cognition, expectations, commitment and perceived resources?

• on the organizational level, concerning content, process and environment?

### Primary outcome measures

Health care consumption, functional abilities and satisfaction with health and social care.

### Secondary outcome measures

Fatigue, physical activity, activities of daily living, cognition, visual impairment, quality of life, life satisfaction, accessibility, social support, falls, fear of falls, decisional autonomy, morbidity and mortality.

### Measurements of frailty indicators

We use the same definitions and measurements of frailty as the study "Elderly persons in the risk zone" [28] which also is part of the research programme "Support for frail elderly persons - from prevention to palliation". A more detailed description of the frailty indicators is given in the study protocol for "Elderly persons in the risk zone" [28].

The following definitions/cut-off levels of frailty are used:

**Weakness: **Reduced grip strength was considered to be below 13 kg for women and 21 kg for men for the right hand, and below 10 kg for women and 18 kg for men for the left hand, using a North Coast dynamometer [29].

**Fatigue**: Answering Yes to the question "Have you suffered any general fatigue/tiredness over the last three months? (part of "The Göteborg quality of life instrument" [30]).

**Weight loss**: Answering Yes to the question "Have you suffered any weight loss over the last three months? (part of "The Göteborg quality of life instrument" [30]).

**Reduces physical activity**: Taking outdoor walks 1-2 walks/week or less.

**Impaired balance**: Having a value of 47 or less on the Berg Balance Scale [31-33].

**Reduced gait speed**: Walking four metres with a gait speed of 0.6 metres/second or slower [34].

**Visual impairment**: Having a visual acuity of 0.5 or less using the KM chart [35].

**Impaired cognition**: Scoring below 25 on the Mini Mental State Examination (MMSE) [36].

### Statistical analysis and power calculation

A power calculation was done before the start of the study. The calculation was based on the expected relative change over time in functional abilities, i.e. we assumed that the intervention group would change slightly or not at all in their functional status and that the control group would deteriorate by 20% in relation to the intervention group (this assumption was based on clinical experience). Power calculations ensured that we would be able to reveal a difference of at least 20% between the groups, if the hypothesis was true. To be able to detect a difference of at least 20% with a two-sided test and with at significance level of alpha = 0.05 and 80% power we needed at least 95 people in each group. Thus a total of approximately 200 people was planned to be included.

An interim analysis was made to evaluate whether or not the assumed difference evaluation was relevant. This was done with knowledge of more specific prevalence rates of functional abilities which, since the study began, have been gained from the study "Elderly persons in the risk zone" [28]. This knowledge enabled us to make a more detailed power calculation. The prevalence rates were for less frail elderly persons than those in our study. Thus we assumed lower functional status and higher standard deviance. This power calculation was based on the balance scale (one of the primary outcome variables, range 0-56), with an assumed mean for the intervention group of 32 and for the control group of 28 (15% difference), and a standard deviation of 8 in both groups. To be able to detect a difference between the intervention and control groups with a two-sided test and with a significance level of alpha = 0.05 and 80% power we would need at least 65 people in each group.

The analysis will be made on the basis of the intention-to-treat principle, meaning that participants will be analysed on the basis of the group to which they were initially randomised [37]. Both descriptive and analytic statistics will be used to compare the two groups as well as for analyses of changes over time. Non-parametric statistics will be used when ordinal data are analysed. Otherwise, parametric statistics will be used.

### Implementation study

The implementation study have a case study design [38] enabling a close study of the actual organisational practices [39]. Multiple methods are used to collect the empirical data, and the material is collected throughout the study. Data collection methods include direct observations, qualitative interviews, questionnaires and project documentation. Direct observations of the work carried out by the case manager and the patient care planning meetings performed by the multi-professional team in the municipality, qualitative interviews with the members of the multi-professional team in the municipality, the geriatric nurses at the emergency ward of the hospital, managers involved in the project at different levels in the municipality, hospital and primary care, questionnaire items regarding the participants as well as their relatives' experience of the intervention programme and project documentation constitute the empirical data of the implementation study. Altogether, this forms a rich empirical material enabling many different analyses.

The process of implementing the intervention programme is analysed from both a bottom-up [40] and a knowledge transference perspective [41,42]. The bottom-up perspective emphasise the actions of the professionals at the operative or street level [43] of the organisations involved as they transform the intervention programme into practice. How they carry out the intervention programme is seen as being affected by the cognitions, expectations, commitment and perceived resources of the street-level staff. From the knowledge transference perspective, implementation problems are foremost seen as problems in bridging the gap between science and practice. It is thus important to identify components that support or inhibit the process of implementing the intervention programme [41,44,45].

The process of implementing the intervention programme is studied both at the operative, management and organisational levels. On the operative level the actions of the professionals involved are in focus, while on the operative and management level the cognition, expectations, commitment and perceived resources are analysed. Programme fidelity, the participants' responsiveness to the programme, and implementation strategies are investigated as well. On the organisational level implementation content, process and environment are analysed, and cultures, inter-organisational linkages, and historical as well as concurrent events are included in a more comprehensive analysis.

### Economic analysis

Cost-utility analyses (CUA) will be used for the economic analysis of the intervention study. In such an analysis, the health effects of the intervention are quantified as quality adjusted life years (QALY). The main outcome of the CUA is the incremental costs per QALY. The incremental cost utility ratio (ICUR) is calculated by comparing the difference between the intervention and control groups in average costs per person with the difference in QALY per person. Health-related quality of life is measured at baseline and all follow ups using the European Quality of Life Instrument (EQ-5D) [46].

### Time plan of the study

The inclusion process began in October 2008 and was completed by the end of June 2010. The intervention began at the same time as the inclusion, and will be completed one year after the last inclusion, that is by the end of June 2011. The follow up after one year will be completed in July 2011. See table 2 for time plan for the study and the follow ups.

**Table 2:** Time plan of the study and follow ups

	Started	Completed	Will be completed in
Inclusion	October 2008	June 2010	
Baseline	October 2008	June 2010	
3 months	January 2009	September 2010	
6 months	April 2009	April 2011	
1 year	October 2009		July 2011

### Baseline characteristics

**Figure 1:**
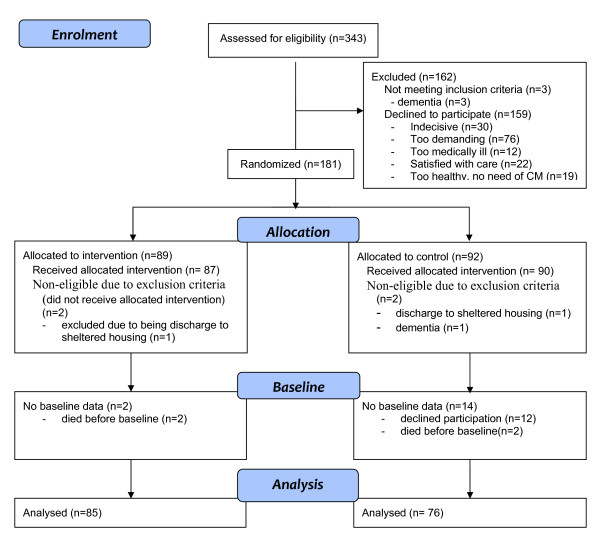
**Flow chart of enrolment, allocation and baseline**.

During the time of inclusion, 1,445 elderly persons living in the municipality of Mölndal sought care at the emergency ward of Mölndal Hospital when the geriatric nurse was at the emergency ward. Of these, 343 met the inclusion criteria, and were therefore invited to participate in the study, see figure 1. See figure 1 for number of persons randomized, receiving allocated intervention, having baseline data and reasons for not participating.

The median age of the participants was 83 in both groups, with the age range of 66-92 in the control group and 70-96 in the intervention group. Visual impairment was more common in the control group. Otherwise there were no statistically significant differences between the groups in terms of baseline characteristics concerning demographic and frailty indicators, see tables 3 and 4. The non-participants had a median age of 78 (range 69-94), and 61% were women.

**Table 3:** Baseline characteristics of study participants

Characteristics	Control group n = 76 %	Intervention n = 85 %	p-value
Female	55.3	55.3	1.00
Living alone	60.5	56.5	0.63
Academic education	15.8	12.2	0.51
Self-rated health (excellent/very good/good)	28.0	40.7	0.10

**Table 4:** Frailty indicators of study participants

Frailty indicators	Control group n = 76 %	Intervention n = 85 %	p-value
Weakness	23.6	21.3	0.85
Fatigue	69.7	72.8	0.73
Physical activity	51.3	46.4	0.63
Weight loss	40.8	35.8	0.62
Gait speed	57.3	51.2	0.52
Poor balance	60.0	54.8	0.52
Visual impairment	85.5	70.6	0.02
Impaired cognition	3.9	9.9	0.21

## Discussion

The study "Continuum of care for frail elderly people" was designed to evaluate whether or not a multi-professional and multidimensional intervention targeting frail elderly persons can decrease health care utilization, maintain functional abilities and increase satisfaction with health and social care. One of the major strengths of this study is that the implementation of the intervention programme is followed and analysed throughout the study. This enables us to generate knowledge on the process of implementing complex intervention in health and social care settings, both in term of how the intervention is perceived and translated into practical clinical work and to see what is in the "black box", i.e. to explore how and why different parts of the intervention were/were not implemented. Another major strength is that it has both an explorative and experimental design, facilitating multi-facetted knowledge production. In addition, it is a randomised controlled study, which is very important for the possibility of drawing valid conclusions from the results.

We were not able to include as many frail elderly people as was at first calculated. Still, the 181 included are sufficient enough according to the more detailed interim power calculation, and should be enough even the inevitable attrition in the follow ups. The non-participation rate, 46.8%, is in line with what can be expected for this very frail elderly group of patients, especially as they had to decide whether or not to participate when at the emergency ward with acute symptoms. The median age of the non-participants was somewhat lower than the participants, but the age range was about the same. The most common reasons for not participating were that the study seemed too demanding. There were also a number of persons who were too healthy to see any reason to participate. Thus, there are indications of the non-participants being both healthier and less healthy than the participants. Therefore, the participants can be seen as a fairly representative sample of the frail elderly population.

This study used almost the same questionnaire, measurements and manuals as the previous study "Elderly persons in the risk zone"[28], for which the outcome measures were selected very carefully to make sure that they had clear psychometric properties, i.e. were valid and reliable for the target group and measured/covered the different components of the frailty concept. The logistics were tested during the pilot study. The fact that we used the same measurements enables us to compare the results from both studies, which further strengthens them. The participants in the present study were clearly frailer than the participants in "Elderly persons in the risk zone", which is obvious if one compares how many of the participants fulfilled the frailty indicators in the studies. For example, only 5-8% had weight loss in "Elderly persons in the risk zone" compared to 35-40% in our study. Overall, there were more participants in our study with frailty indicators, but the distribution was similar, with visual impairment and fatigue being the most prevalent indicators in both studies and weakness and weight loss being less prevalent. The fact that impaired cognition was rare can be explained by dementia/severe cognitive impairment being an exclusion criterion. Visual impairment was the only variable that was statistically significantly different between the intervention and the control group. Since the allocation was randomized, this ought to be due to mere chance. 28-40% of the participants rated their health as excellent/very good/good, compared to about 80% in "Elderly persons in the risk zone". Thus, the participants in this study are probably representative of the frail elderly population.

We were not able to keep the research assistants blinded to group assignment, which is a major limitation of the study. On the other hand, there were advantages to the research assistant knowing the assignment, such as ensuring that all participants received the intervention could report their experiences of it, and lowering the attrition between follow ups thanks to personal contact between the interviewer and the elderly person. Another limitation is that the interviewers had different professional backgrounds. To strengthen the reliability, the interviewers were trained in using the questionnaire, the different measurements and the manual. To further strengthen validity, protocol meetings were held throughout the study. The different professional backgrounds of the interviewers can also be argued to strengthen the study, since they benefitted from their colleagues' competence and knowledge.

The intervention was planned and elaborated in collaboration with representatives of the different care levels included in the intervention, i.e. emergency ward, department of geriatrics, department of internal medicine, municipal health and social care, and primary care. Regular meetings to discuss the content of the intervention, inclusion/exclusion criteria, measurements and logistics were held during both the planning and intervention periods. This enhances the implementation and strengthens the study. The intervention includes many different aspects previously shown to have positive effects on the target group, such as multidisciplinary and multi-factorial, individualized assessment and intervention, case management, comprehensive geriatric assessment, geriatric screening at the emergency ward and home based team intervention. In conjunction with the implementation process, this enables us to analyse what parts of the intervention contributes to a positive outcome. The research team as well as the group of professionals carrying out the intervention are multi-professional. We consider this essential to performing such a complex intervention. It ensures the multi-dimensionality of the study which is needed in both performing the study and in interpreting the results.

In summary, the intervention - including an early geriatric assessment, early family support, a case manager in the community with a multi-professional team and involvement of the elderly people and their relatives in the planning process - contributes to early recognition of the elderly peoples' needs of information, care and rehabilitation and of informal caregivers' need of support and information. An intervention creating a continuum of care for frail elderly people can have many advantages, both in terms of health and economics, for the individual as well as for society. It enhances the transfer of information and integrates the care between different caregivers and different care levels, thereby better recognizing frail elderly peoples' needs. Specifically, this study is expected to show positive effects of the multi-dimensional and multi-professional intervention on the frail elderly peoples' health care consumption, functional abilities and satisfaction with health and social care.

## Competing interests

The authors declare that they have no competing interests.

## Authors' contributions

KW led the intervention of the study, and was the primary author of the manuscript. KW, AD, KE, GGH and SB participated in the research design and implementation of the study. AD, SB and HH has followed and analysed the implementation process. KW, AD, KE, GGH and SDI contributed to the aim and the outcome measurements. HG has participated in the implementation of the study. SL and SDI participated in the research design of the study. SDI led the research design of the study. All authors contributed to the writing and review of the manuscript and approved of the final manuscript.

## Pre-publication history

The pre-publication history for this paper can be accessed here:

http://www.biomedcentral.com/1471-2318/11/24/prepub
